# Comparison of EUS and ERCP-guided tissue sampling in suspected biliary stricture

**DOI:** 10.1371/journal.pone.0258887

**Published:** 2021-10-20

**Authors:** Hye Gyo Chung, Jong-In Chang, Kwang Hyuk Lee, Joo Kyung Park, Kyu Taek Lee, Jong Kyun Lee

**Affiliations:** Department of Medicine, Samsung Medical Center, Sungkyunkwan University School of Medicine, Seoul, Korea; Zhejiang University, CHINA

## Abstract

**Background:**

Endoscopic retrograde cholangiopancreatography (ERCP) and endoscopic ultrasound (EUS) are commonly used diagnostic modalities in biliary strictures. We compared the diagnostic yield of EUS and ERCP-based tissue sampling in intrinsic biliary strictures without extrinsic mass outside the bile duct.

**Methods:**

A total of 85 patients who underwent ERCP and EUS for diagnosis of suspected biliary strictures confined to the bile duct were analyzed retrospectively at Samsung Medical Center, Seoul, Korea, between 2010 and 2018.

**Results:**

Seventy-one patients were diagnosed with malignancy and 14 patients were diagnosed with benign strictures. EUS-based tissue sampling was more sensitive and accurate than ERCP-based tissue sampling (p = 0.038). The overall sensitivity and accuracy were 67.6% (95% confidence interval (CI) 56.1–77.3) and 72.9% (95% CI 62.7–81.2) for ERCP-based sampling, and 80.3% (95% CI 69.6–87.9) and 83.5% (95% CI 74.2–89.9) for EUS-based sampling, respectively. EUS-based sampling was superior to ERCP-based sampling in distal bile duct strictures (accuracy: 87.0% vs. 72.5%, p = 0.007), but not in perihilar strictures. In cases without intraductal mass, EUS-based tissue sampling was also superior to ERCP-based sampling (accuracy: 83.3% vs. 69.7%, p = 0.029), but not in cases with mass.

**Conclusion:**

EUS-based tissue sampling was superior to ERCP-based method in intrinsic biliary stricture with no mass outside the bile duct, particularly in those without intraductal mass or those with strictures located in distal bile duct. Therefore, EUS-based sampling should be considered for making a pathological diagnosis of suspected distal bile duct strictures even in lesions without definite mass.

## Introduction

Diagnosis of suspected biliary stricture is challenging, as the differential diagnosis includes cholangiocarcinoma, pancreatic cancer, ampulla of vater (AoV) cancer, metastatic cancer of other primary malignancies as well as benign biliary strictures [1]. Previous studies have reported 55–95% malignancy rates in patients with suspected biliary stricture [2–6]. Abdominal ultrasound, computed tomography scan, and magnetic resonance imaging provide information on structural abnormalities; however, they cannot be used for definitive diagnosis in patients with biliary strictures.

Today, cytopathologic diagnosis of biliary tract cancer with endoscopic approaches is commonly used, with the main ones being ERCP-based tissue sampling and EUS-guided fine needle aspiration/biopsy (EUS-FNA/B). ERCP has been widely used for tissue sampling with a sensitivity of 35–75% in suspected biliary obstruction [3,4,7,8]. Bile duct obstruction can simultaneously be treated with balloon dilatation or stent placement during ERCP, making it an attractive modality. As a newer method of tissue sampling, EUS has a sensitivity of 50–95% in suspected biliary obstruction [3–5,9–11]. In a limited number of studies which compared the diagnostic performances of EUS and ERCP-based tissue sampling, the EUS-based method was better than ERCP-based sampling [4,6,10,12–15]. However, the patient populations in these studies primarily consisted of those with extraductal lesions like pancreatic cancers, from which the EUS-based technique seemed to be theoretically more suitable for getting tissue samples. For this reason, it is not clear which modality is better in biliary strictures, in which the lesion is confined to the bile duct. In this study, we aimed to compare the diagnostic yield of EUS and ERCP-based tissue sampling in biliary strictures not accompanied by mass in adjacent organs.

## Materials and methods

### Study setting, design, patients

**Fig 1:**
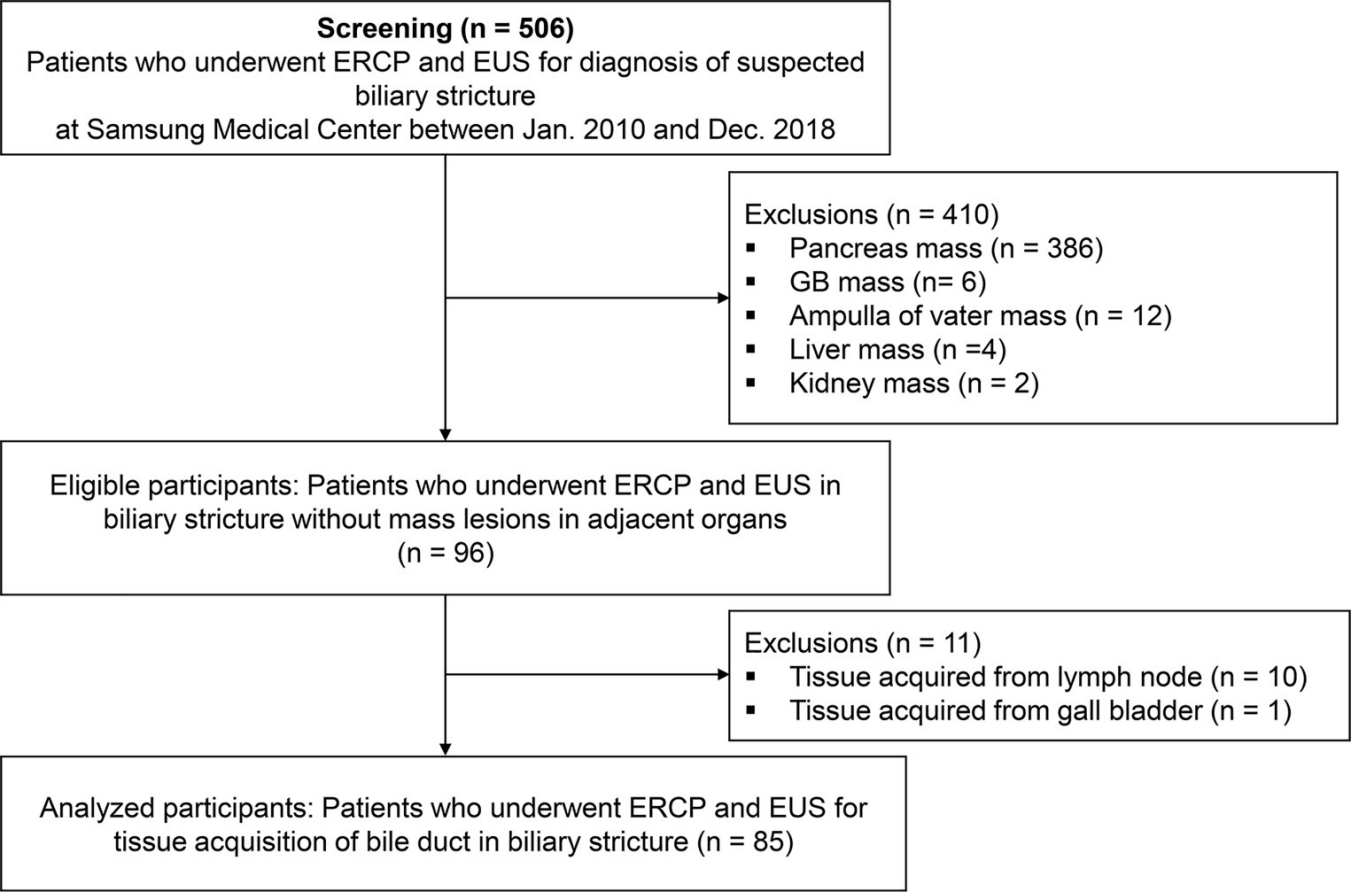
Flowchart of the study.

Five hundred and six patients underwent both ERCP and EUS for the diagnosis of suspected biliary stricture at Samsung Medical Center, Seoul, Korea, between January 2010 and December 2018. Suspicion of biliary stricture was based on clinical history, imaging studies including abdominal computed tomography (CT), and magnetic resonance imaging (MRI). Biliary stricture was suspected when dilated upstream bile ducts with or without thickened bile duct, obstructive mass, abnormal liver function tests and/or jaundice. We classified biliary strictures into two categories: extrinsic and intrinsic. An extrinsic biliary stricture was defined as a stricture caused mainly due to extrinsic compression from masses associated with the pancreas, gall bladder, liver or lymph node. These can be targeted with EUS, as the anatomopathology is mainly located outside the biliary tract. Therefore, we included in the study only those defined as intrinsic biliary stricture without mass or with mass confined to bile duct which could be accessed through bile duct lumen with ERCP. We excluded patients under the age of 18 and patients whose tissue and cytology samples were acquired from lymph nodes, ampulla of vater, or organs other than bile ducts during EUS. A total of 85 patients were included for the final analysis (Fig 1). We reviewed the medical, endoscopic, histopathologic, and radiographic records with the approval of the Institutional Review Board at Samsung Medical Center (IRB No. SMC 2020-01-127-001). Medical records provided relevant information-including demographic variables (age, gender), initial symptoms and signs of patients, laboratory results, and follow-up clinic visits. Patients’ medical records/samples were accessed by the study staffs between Nov 2019 and Feb 2020. As the study used only de-identified data routinely collected during admission and hospital visits, the requirement of obtaining informed consent from the patients was waived.

### Radiologic imaging

Patients underwent CT and/or MRI for initial evaluation preceding either ERCP or EUS exam. The location of the mass or stricture lesion was designated perihilar or distal based on a prior study [16]. Perihilar location was defined as those involving hepatic bifurcation without a significant intrahepatic component, and distal location was defined as those involving distal extrahepatic, or intrapancreatic portions of the bile duct.

### ERCP/EUS examination

ERCP and EUS examinations were performed under conscious sedation by highly experienced investigators each of whom performs more than 300 EUS and ERCP procedures each year. ERCP and EUS examinations were performed either on the same day or less than a few days apart at the discretion of the primary physician, before the pathology and/or cytology results were confirmed for the preceding exam.

For ERCP, a duodenoscope (TJF-260; Olympus Medical Systems, Tokyo, Japan) was used. After cannulation and cholangiography, sphincterotomy or balloon dilatation was performed in most cases. The brush (RX Cytology Brush Wireguided Cytology Brush, Boston Scientific) was inserted into the bile duct over the guidewire (0.025- or 0.035-inch Visiglide; Olympus). Brushing cytology was obtained with more than 10 to-and-fro brushing strokes passing the biliary stricture. The brush was smeared onto the slide, which was fixed in alcohol and transferred to the pathology department. Intraductal biopsy was done with forceps (Single-Use Radial Jaw™ 4 Biopsy Forceps, Boston Scientific or Reusable Biopsy Forceps, Olympus Medical Systems) directly through the AoV with fluoroscopic guidance.

We used a linear echoendoscope for EUS examination. Following EUS evaluation, we punctured the suspected biliary stricture lesion. More than two FNA passes were made using 19, 22 or 25-gauge EchoTip® Needle (Wilson-Cook Inc., Winston-Salem, NC, USA) or Acquire® Needle (Boston Scientific Co., Marlborough, MA, USA) (S1 Table). We tried to use the larger bore needle as possible. But if the position of endoscope didn’t allow the large bore needle, we did EUS-FNA with next smaller diameter needle. In most cases, the needle was moved back and forth at least 10 times and negative suction was applied. Procedures were done without the presence of an on-site cytopathologist.

Technical failure was defined as failure of the intended EUS or ERCP guided tissue sampling. The case was regarded as a technical failure when the lesion was visualized on EUS but safe puncture of the lesion was not feasible due to intervening vessels, pancreatic duct and so on.

Tissue and cytology samples were classified as follows; (1) malignant; (2) atypical, suspect malignant; (3) atypical cells of undetermined significance; (4) atypical, suspect benign; (5) benign; (6) non-diagnostic, insufficient sample. Of those, “malignant” and “atypical, suspect malignant” were considered to be positive for malignancy. “atypical, suspect benign” and “benign” were considered as negative for malignancy. “atypical cells of undetermined significance” were counted as non-diagnostic. For cases of failed acquisition of tissue or cytology samples, these were regarded as non-diagnostic by intention-to-treat analysis. When surgical resection was performed after endoscopic exams, histopathology was considered to be the reference standard for the presence of malignancy. In other cases of nonoperative management with nondiagnostic pathology and cytology reports, subsequent clinical courses including image studies with more than six months of follow-up were considered to be the reference standard for the diagnosis. All patients with negative tissue samples were followed-up for more than six months with image studies to reduce the risk of false negative result.

### Statistical analysis

Continuous variables were expressed as mean values with standard deviation or median with ranges. Categorical variables were expressed as counts with percentages. To determine the operating characteristics of EUS-FNA/B and ERCP-based sampling in diagnosing the cause of biliary stricture, the sensitivity, specificity, positive predictive value, negative predictive value, accuracy, and their corresponding 95% confidence intervals (CIs) were calculated using Wilson’s method [17]. Results were compared between EUS-FNA/B and ERCP-based sampling using chi-square test and McNemar test [18]. The McNemar test was used to compare the sensitivity, specificity, and accuracy of two diagnostic modalities and the chi-square test was used to compare positive predictive value and negative predictive value. A p value less than 0.05 was regarded as statistically significant. All statistical analyses were performed with Statistical Analysis System 9.4 software (SAS INC., Cary, NC, USA).

## Results

### Patient demographic and clinical characteristics

Demographic and clinical characteristics for the 85 patients analyzed in the study are shown in Table 1. Stricture alone was observed in 77.6% (66/85) of patients and stricture accompanying mass within the bile duct was identified in 19 (22.4%) patients on CT and/or MRI. Sixteen (18.8%) patients had biliary stricture in perihilar location and the other 69 (81.2%) patients had lesions in distal bile duct. Hyperbilirubinemia and abnormal liver function test were documented in 59 (69.4%), and 72 patients (84.7%) respectively, at initial presentation. Six (7.1%) patients were suspected to have biliary stricture in imaging studies without having any symptoms. Seventy-seven (90.6%) patients underwent EUS and ERCP simultaneously and 8 (9.4%) patients received the procedures on a different day before the cytopathology of the preceding exam was confirmed.

**Table 1:** Baseline characteristics of patients.

Variable	Overall (n = 85)
Age, years	68 (28–84)
Gender: male/female, n (%)	52 (61.2)/33 (38.8)
CT and/or MRI evaluation	
Biliary stricture with intraductal mass	19 (22.4)
Biliary stricture alone	66 (77.6)
Location	
Perihilar	16 (18.8)
Distal	69 (81.2)
Symptoms and signs	
No specific symptoms	6 (7.1)
Hyperbilirubinemia	59 (69.4)
Abdominal pain	34 (40.0)
Weight loss	9 (10.6)
Abnormal liver function test	72 (84.7)
Size of target lesion (mm)	19.0 (5.0–46.0)
Final diagnosis	
Malignant	71 (83.5)
Cholangiocarcinoma	70 (98.6)
Metastatic gastric cancer	1 (1.4)
Benign	14 (16.5)
Biliary intraepithelial neoplasia, low grade	2 (14.3)
Idiopathic benign biliary stricture	10 (71.4)
Autoimmune pancreatitis	1 (7.1)
Chronic pancreatitis	1 (7.1)
Method of final diagnosis	
Surgical pathology	56 (65.8)
EUS and/or ERCP	25 (29.4)
Pathologic or cytologic confirmation by other method	2 (2.4)
Clinical assumption (follow up > 6 months)	2 (2.4)

Numeric variables are expressed as mean ± standard deviation or median (range). Categorical variables are expressed as number (percentage).

### Final diagnosis and methods

For the final diagnosis, malignancy was confirmed in 71 (83.5%) patients (Table 1). Among those, 70 (98.6%) were diagnosed with cholangiocarcinoma and 1 (1.4%) had metastatic gastric cancer. The other 14 (16.5%) were diagnosed with benign strictures, two of which were low grade biliary intraepithelial neoplasia and 10 patients were diagnosed with idiopathic benign biliary strictures that were diagnosed when there was no history of trauma, surgery and stones, no pathology suggesting IgG4 and PSC and no evidence of infections including parasites.

In 23 cases of negative samples from both EUS and ERCP, each case was discussed by a multidisciplinary team, which consisted of endoscopists, surgeons, radiologists, and pathologists. In case the possibility of malignancy was high and the lesions could be resected completely, surgery was offered. Nine patients underwent surgery, confirming five malignant and four benign strictures. In case the possibility of benign stricture was high, repeated endoscopy was recommended. Six patients underwent repeated endoscopies confirming benign strictures. The other four patients who refused repeat endoscopies were followed up periodically and were determined to have benign strictures. In four cases with possibly malignant but inoperable lesions, close follow-up with additional diagnostic methods were tried.

There were nine patients whose initial EUS and ERCP-based tissue sampling was negative for malignancy yet were diagnosed with cholangiocarcinoma upon additional tests. Five patients (5.9%) were diagnosed with cholangiocarcinoma by surgical pathology and there were two cases (2.4%) of cholangiocarcinoma diagnosed following bile cytology or liver biopsy. Two patients (2.4%) were followed up for more than six months and diagnosed based on deteriorating clinical course including imaging studies (Table 1). For those whose pathology and/or cytology samples of ERCP and EUS were positive for malignancy, there were no diagnostic discrepancies when compared with the surgical pathology. Patients who were diagnosed to have malignancies were managed according to cancer stages except for 14 patients who received supportive cares only mostly due to old age and medical comorbidities (S2 Table).

The technical success rate of EUS-based tissue sampling was 87.1% (Table 2). In eleven cases of technical failure, the lesions were visualized on EUS but safe puncture was not feasible. Of the 11 patients with failed EUS-based tissue sampling, the final diagnosis was confirmed by surgical pathology of bile ducts (two patients), ERCP-based biopsy (eight patients) and liver biopsy during follow-up (one patient). For those with technical success, both cytology and biopsy samples were acquired in 62 (83.8%) patients, cytology samples alone in 10 (13.5%) and biopsy samples alone in two (2.7%).

**Table 2:** Results of EUS and ERCP-based tissue sampling.

EUS		
Technical success	74 (87.1)	
Acquired specimen		
Cytology	10 (13.5)	
Pathology	2 (2.7)	
Cytology with pathology	62 (83.8)	
Needle size		
19G	2 (2.8)	
22G	36 (48.6)	
25G	36 (48.6)	
ERCP		
Technical success	80 (94.1)	
Biliary stent insertion	74 (87.1)	
Acquired specimen		
Intraductal biopsy	17 (21.3)	
Brushing cytology	34 (42.5)	
Intraductal biopsy with brushing cytology	29 (36.2)	
	EUS (n = 74)	ERCP (n = 80)
Malignant	53 (71.6)	44 (55.0)
Atypical, suspect malignant	4 (5.4)	4 (5.0)
Atypical cells of undetermined significance	1 (1.4)	2 (2.5)
Atypical, suspect benign	5 (6.8)	3 (3.75)
Benign	10 (13.5)	27 (33.75)
Non-diagnostic	1 (1.4)	

Categorical variables are expressed as number (percentage).

ERCP was technically successful in 94.1%. Failure of cannulation was the main reason for technical failure. For those five patients with technical failure, pathologic diagnosis was confirmed by surgical pathology of bile duct (one patient) and EUS-based sampling (four patients). For those with technical success, both intraductal biopsy and brushing cytology were performed in 29 (36.2%) patients, intraductal biopsy alone in 17 (21.3%), and brushing cytology alone was performed in 34 (42.5%).

#### Diagnostic performance and adverse events of EUS and ERCP-based sampling

The overall sensitivity and accuracy were 67.6% (95% CI 56.1–77.3), and 72.9% (95% CI 62.7–81.2) for ERCP-based sampling and 80.3% (95% CI 69.6–87.9), and 83.5% (95% CI 74.2–89.9) for EUS-guided sampling, respectively. EUS-based tissue sampling was superior to ERCP-based sampling (p = 0.038, Table 3).

**Table 3:** Diagnostic performance of EUS and ERCP-based tissue sampling (n = 85).

	ERCP	EUS	p value
Sensitivity (95% CI), %	67.6 (56.1–77.3)	80.3 (69.6–87.9)	0.038
Specificity (95% CI), %	100 (78.5–100)	100 (78.5–100)	
Positive predictive value (95% CI), %	100 (92.6–100)	100 (93.7–100)	
Negative predictive value (95% CI), %	37.8 (24.1–53.9)	50.0 (32.6–67.4)	0.07
Accuracy (95% CI), %	72.9 (62.7–81.2)	83.5 (74.2–89.9)	0.038

In the subgroup without an intraductal mass, the sensitivity and accuracy were 62.3 (95% CI 48.8–74.1), and 69.7 (95% CI 57.8–79.5) for ERCP and 79.3 (95% CI 66.5–88.0) and 83.3 (95% CI 72.6–90.4) for EUS, respectively, showing superiority of EUS (p = 0.029, Table 4). However, in the subgroup with an intraductal mass, the sensitivity and accuracy were 83.3% (95% CI 60.8–94.2) and 84.2% (95% CI 62.4–94.5) for ERCP and 83.3% (95% CI 60.8–94.2) and 84.2 (95% CI 62.4–94.5)) for EUS, demonstrating no difference in diagnostic performances (p = 1.00).

**Table 4:** Subgroup analysis of diagnostic performance of EUS and ERCP according to the presence of intraductal mass.

		ERCP	EUS	p value
Stricture (n = 66)	Sensitivity (95% CI), %	62.3 (48.8–74.1)	79.3 (66.5–88.0)	0.029
	Specificity (95% CI), %	100 (77.2–100)	100 (77.2–100)	
	Positive predictive value (95% CI), %	100 (89.6–100)	100 (91.6–100)	
	Negative predictive value (95% CI), %	39.4 (24.7–56.3)	54.2 (35.1–72.1)	0.06
	Accuracy (95% CI), %	69.7 (57.8–79.5)	83.3 (72.6–90.4)	0.029
Mass (n = 19)	Sensitivity (95% CI), %	83.3 (60.8–94.2)	83.3 (60.8–94.2)	1.00
	Specificity (95% CI), %	100 (20.7–100)	100 (20.7–100)	
	Positive predictive value (95% CI), %	100 (79.6–100)	100 (79.6–100)	
	Negative predictive value (95% CI), %	25.0 (4.6–69.9)	25.0 (4.6–69.9)	1.00
	Accuracy (95% CI), %	84.2 (62.4–94.5)	84.2 (62.4–94.5)	1.00

When analyzed further according to the location of lesion, the sensitivity and accuracy were 66.7% (95% CI 53.0–77.1), and 72.5% (95% CI 61.0–81.6) for ERCP and 83.9% (95% CI 72.2–91.3) and 87.0% (95% CI 77.0–93.0) for EUS, respectively, in strictures located in distal common bile duct (p = 0.007, Table 5). However, the sensitivity and accuracy between two groups were not different in hilar strictures.

**Table 5:** Subgroup analysis of diagnostic performance of EUS and ERCP according to biliary stricture location.

		ERCP	EUS	p value
Perihilar (n = 16)	Sensitivity (95% CI), %	73.3 (48.1–89.1)	66.7 (41.7–84.8)	0.65
	Specificity (95% CI), %	100 (20.7–100)	100 (20.7–100)	
	Positive predictive value (95% CI), %	100 (74.1–100)	100 (72.3–100)	
	Negative predictive value (95% CI), %	20.0 (3.6–62.5)	16.7 (3.0–56.4)	0.68
	Accuracy (95% CI), %	75.0 (50.5–89.8)	68.8 (44.4–85.8)	0.65
Distal (n = 69)	Sensitivity (95% CI), %	66.7 (53.0–77.1)	83.9 (72.2–91.3)	0.007
	Specificity (95% CI), %	100 (77.2–100)	100 (77.2–100)	
	Positive predictive value (95% CI), %	100 (90.6–100)	100 (92.4–100)	
	Negative predictive value (95% CI), %	40.6 (25.5–57.7)	59.1 (38.7–76.7)	0.031
	Accuracy (95% CI), %	72.5 (61.0–81.6)	87.0 (77.0–93.0)	0.007

Procedure-related adverse events were documented in 14 (16.5%) patients, all of which were post-procedure acute pancreatitis managed with intravenous hydration and fasting for 1–2 days. There were no cases of bleeding or bowel perforation.

## Discussion

Accurate pathologic and cytologic diagnosis of presumed biliary stricture before surgical resection remains challenging. ERCP has been widely used for diagnosis due to the convenience and advantages of simultaneous stent placement. EUS is a relatively new method particularly well suited for evaluation of anatomical structures around the bile duct [19–21]. To identify better sampling methods, there have been studies which compared diagnostic yields for ERCP and EUS in suspected biliary stricture [4,6,10,12–14]. A prospective study of 51 patients with suspected malignant biliary obstruction from the United States [4], a multicenter retrospective study consisting of 263 patients from Korea [12], and a retrospective study with a series of 37 patients in United Kingdom [10] have reported better overall diagnostic yield of EUS-based sampling compared to ERCP-based tissue sampling. However, the vast majority of tissue samples taken were from masses around the bile duct (e.g. pancreas), readily approachable with EUS but not with ERCP in most cases. In lesions with biliary stricture alone, on the other hand, the diagnostic yield of ERCP-based sampling was comparable to or better than that of EUS-guided tissue sampling. To our knowledge, no previous study has investigated the diagnostic performances of EUS and ERCP in suspected biliary strictures in details, focusing especially on strictures due to intrinsic causes. In this study, we compared the diagnostic performances of EUS and ERCP-based sampling in cases of presumed intrinsic biliary strictures with no proven mass lesions in adjacent organs based on imaging studies such as CT scans or MRI. Overall, EUS-based tissue sampling demonstrated greater sensitivity and accuracy at 80.3% and 83.5% respectively, which is higher than seen with ERCP-based sampling, at 67.6% and 72.9%, respectively. When stratified according to the presence of intraductal mass and the location of strictures, EUS-based tissue sampling was superior to ERCP-based sampling in biliary strictures with no intraductal mass lesions and in distal strictures. It is likely that EUS, with its advanced imaging technique, can visualize the thickened wall of bile duct without definite intraductal mass lesions on CT or MRI thus making tissue sampling feasible with a targeted approach. EUS exhibits superiority over ERCP-based tissue sampling in this population. In biliary strictures with an accompanying intraductal mass on the other hand, ERCP has advantages in targeting and acquiring tissue samples as it is performed with fluoroscopic guidance.

As existing literature has indicated, EUS was particularly useful in diagnosing distal bile duct strictures [22,23]. This could be attributed to the anatomical location of bile duct in that the distal portion lies near the duodenal wall where the EUS transducer is placed. Yet, our results are inconsistent with previous studies in which EUS-based sampling was only comparable with or inferior to ERCP in lesions with biliary strictures alone without intraductal mass [3,4,12]. However, in these studies the majority of the enrolled patients had pancreatic masses and the tissue samples of earlier studies were taken from sites other than bile ducts (e.g., Pancreas, lymph nodes) as well. We believe differences in the population of enrolled patients and samples acquired from different sites other than bile ducts might explain these discrepancies. A prospective study with a large number of patients would validate our findings.

The overall negative predictive value was 50.0% for EUS-FNA/B and 37.8% for ERCP-based sampling (Table 3). The negative predictive value in 16 patients with perihilar bile duct stricture was 16.7% for EUS-based sampling and 20.0% for ERCP-based sampling (Table 5). This indicates that other diagnostic approach methods should be encouraged when initial cytopathology of EUS and ERCP-based sampling is negative for malignancy despite a strong clinical suspicion of malignancy.

EUS-FNA/B for perihilar cholangiocarcinoma has not been widely encouraged as there is a risk of tumor seeding [24]. The American Gastroenterological Association and American Society for Gastrointestinal Endoscopy currently advise that FNA of perihilar biliary strictures be used with caution, especially when the patient is eligible for liver transplantation [25,26]. However, a large cohort study consisting of 150 patients with cholangiocarcinoma in the United States revealed that preoperative EUS-FNA did not have a negative impact on overall survival or progression-free survival. There was no difference in survival and postoperative recurrence in patients with hilar cholangiocarcinoma specifically [27]. When used with caution, EUS-FNA could be safely performed for tissue acquisition. In addition, various types of peroral cholangioscopies and related accessories are currently available and they have improved to get intraductal tissues even beyond the hilar ducts in some centers[28,29]. Therefore, peroral cholangioscopy should also be considered as a useful diagnostic tool for indeterminate biliary strictures which are thought to be due to intraductal lesions.

This study bears some limitations. We conducted a single center study with retrospective analysis. In addition, the number of enrolled patients was relatively small, particularly for perihilar lesions. The long duration of our study period, i.e. nine years, could be another limitation. Since the imaging quality and the devices used for aspiration and biopsy have improved in recent years, a prospective study would lead to more robust conclusions. Like most studies about EUS-FNA and ERCP cytology accuracy, this study also used gold standard of final diagnoses with surgical pathology, clinical follow up and pathology itself of EUS-FNA or ERCP [3,4,11,30]. Therefore, there was a little chance not to exclude the false positive and negative completely. In attempt to eliminate the possibility of false negative results, repeated ERCP and/or EUS were performed in some patients whose pathology and cytology results were both negative for malignancy. However, this still could have caused a bias and may be addressed by conducting a prospective study. Pathologists were not blinded, and some might argue that this is a confounding element as they had access to the alternative sample. However, pathologic diagnosis was solely performed without being affected by the alternative specimen. Our study also has some strengths. This is, to our knowledge, the first study that compared the diagnostic performance of EUS and ERCP in intrinsic biliary strictures without mass lesions in adjacent organs. Previously published studies mostly comprised patients with pancreatic masses and included very few patients with bile duct strictures only. We also excluded patients whose tissue or cytology samples were taken from sites other than bile duct for direct and accurate comparison. The sensitivity and accuracy of our EUS-based sampling was similar to that reported in existing literatures even though we only included tissues acquired from bile duct, and there was no on-site cytopathologist available. Advanced technology in EUS imaging and devices paired with expert skills might explain these results.

In conclusion, we have found that EUS-based tissue sampling was superior to ERCP in evaluating intrinsic biliary strictures. The sensitivity and accuracy of EUS were both substantially higher in distal bile duct strictures and in strictures without mass formation along the bile duct. Our findings indicate that EUS-based tissue sampling should be considered for making pathological diagnosis of suspected bile duct strictures, especially in lesions without intraductal mass or in distal bile duct lesions, which requires a prospective study with a large number of patients to make more robust conclusions.

## Supporting information

S1 Table Needle types used for EUS-FNA/B. (DOCX)

S2 Table Detailed clinical managements after the pathology/cytology results of ERCP and EUS. (DOCX)
