# Supplementary material for: Comparison of EUS and ERCP-guided tissue sampling in suspected biliary stricture
Source: PLoS One. 2021 Oct 20;16(10):e0258887. doi: 10.1371/journal.pone.0258887 (PMC8528314; doi:10.1371/journal.pone.0258887)
Supplement: S1 Table — (DOCX) [file pone.0258887.s001.docx]

**Supplementary data:**

Comparison of EUS and ERCP-guided tissue sampling in suspected biliary stricture

**S1 Table. Needle types used for EUS-FNA/B**

|  | Echotip®† | Acquire®‡ |
| --- | --- | --- |
| 19G | 2 (2.7) |  |
| 22G | 18 (24.3) | 18 (24.3) |
| 25G | 25 (33.8) | 11 (14.9) |

Categorical variables are expressed as number (percentage).

†. EchoTip® Needle (Wilson-Cook Inc., Winston-Salem, NC, USA)

‡. Acquire® Needle (Boston Scientific Co., Marlborough, MA, USA)
