# Supplementary material for: Comparison of EUS and ERCP-guided tissue sampling in suspected biliary stricture
Source: PLoS One. 2021 Oct 20;16(10):e0258887. doi: 10.1371/journal.pone.0258887 (PMC8528314; doi:10.1371/journal.pone.0258887)
Supplement: S2 Table — (DOCX) [file pone.0258887.s002.docx]

**Supplementary data:**

Comparison of EUS and ERCP-guided tissue sampling in suspected biliary stricture

**S2 Table. Detailed clinical managements after the pathology/cytology results of ERCP and EUS**

| Final Diagnosis | Pathology/cytology result of EUS for malignancy | Pathology/cytology result of ERCP for malignancy | Number of patients | Management |
| --- | --- | --- | --- | --- |
| Malignancy (n=71) | Positive | Negative | 14 (19.7) | A |
|  | Negative | Positive | 5 (7.0) | A |
|  | Positive | Positive | 43 (60.6) | A |
|  | Negative | Negative | 9 (12.7) | B |
| Benign (n=14) | Negative | Negative | 14 (100) | C |

Categorical variables are expressed as number (percentage).

1. All were treated according to cancer stages such as surgery, chemotherapy and supportive care except for those who received supportive care only mostly due to old age and medical comorbidities.
2. Five patients had surgical resections. Biopsies of other sites confirmed malignancies in two cases and progression of the lesions were observed in two cases.
3. Four patients underwent surgical resections without retrying pathologic diagnosis. The others including six patients who underwent repeat ERCP/EUS were followed up for more than 6 months without any changes.
